# Cepharanthine Induces Oxidative Stress and Apoptosis in Cervical Cancer via the Nrf2/Keap1 Pathway

**DOI:** 10.3390/antiox14111324

**Published:** 2025-11-01

**Authors:** Ya-Hui Chen, Jyun-Xue Wu, Shun-Fa Yang, Tze-Ho Chen, Yun-Chia Wu, Tzu-Chi Lin, Yi-Hsuan Hsiao

**Affiliations:** 1 Women’s Health Research Laboratory, Changhua Christian Hospital, Changhua 50006, Taiwan; 106317@cch.org.tw (Y.-H.C.); 183726@cch.org.tw (J.-X.W.); 2 Institute of Medicine, Chung Shan Medical University, Taichung 40201, Taiwan; ysf@csmu.edu.tw; 3 Department of Medical Research, Chung Shan Medical University Hospital, Taichung 40201, Taiwan; 4 Department of Obstetrics and Gynecology, Changhua Christian Hospital, Changhua 50006, Taiwan; 46305@cch.org.tw (T.-H.C.); 182890@cch.org.tw (Y.-C.W.); 5 School of Medicine, Chung Shan Medical University, Taichung 40201, Taiwan; 6 College of Medicine, Kaohsiung Medical University, Kaohsiung 807378, Taiwan; 7 Department of Post-Baccalaureate Medicine, College of Medicine, National Chung Hsing University, Taichung 40227, Taiwan

**Keywords:** cervical cancer, cepharanthine, anticancer activity, apoptosis, ROS, Nrf2/Keap1 pathway

## Abstract

Cervical cancer ranks as a primary contributor to cancer-related deaths in women globally and is the fourth most prevalent malignant neoplasm. Cepharanthine, a naturally occurring biscoclaurine alkaloid extracted from *Stephania cepharantha*, has demonstrated anticancer and antimetastatic efficacy across multiple cancer types. However, its mechanism of action in cervical cancer remains unexplored. Our results demonstrated that cepharanthine effectively suppressed the proliferation and motility of the CaSki, HeLa, and C33A cell lines. Furthermore, cepharanthine triggered apoptosis through Bcl-2 suppression and increased cleaved-PARP-1, Bax, and cleaved-caspase-3 expression and AMPK/p53 phosphorylation, while inducing G0/G1 phase arrest in CaSki cells and sub-G1 phase arrest in HeLa and C33A cells. Additionally, cepharanthine reduced the mitochondrial membrane potential (∆ψm), compromised mitochondrial functionality, and increased reactive oxygen species (ROS) accumulation, promoting oxidative stress via the modulation of the Nrf2/Keap1 pathway in CaSki, HeLa, and C33A cells, which exhibit an anti-cervical cancer effect. Similarly, cepharanthine markedly reduced tumor progression in C33A BALB/c nude mice, which aligns with the in vitro observations. Collectively, these findings indicate that cepharanthine has potential therapeutic applications in the treatment of cervical cancer and warrants future clinical investigation.

## 1. Introduction

Globally, cervical cancer is one of the most common cancers and the fourth leading cause of cancer-related death among women. Statistical data from 2022 indicate approximately 661,000 newly diagnosed cases, with an estimated 348,000 fatalities worldwide [1]. Therapeutic interventions are determined on the basis of multiple factors, including cancer stage, histological type, patient age, overall health status, and individual preferences. Primary treatment modalities include surgical intervention, radiotherapy, chemotherapeutic agents, targeted therapeutics, and immunological approaches, either as monotherapy or in combination [2,3]. Patients presenting with persistent, recurrent, or metastatic cervical carcinoma generally experience unfavorable outcomes, demonstrating limited responsiveness to platinum-based chemotherapy regimens and median overall survival rates of only 7–12 months [4,5,6]; therefore, developing therapeutic strategies and preventing recurrence are critical in the management of cervical cancer.

Cepharanthine (C_37_H_38_N_2_O_6_), a naturally occurring biscoclaurine alkaloid extracted from *Stephania cepharantha*, has demonstrated significant therapeutic potential. In Japanese medical practice, this compound has been utilized for over seven decades to treat various conditions, including radiation-induced leukopenia, alopecia areata, and inflammatory conditions. Recent research has expanded its potential to include antiviral, anti-inflammatory, immunomodulatory, and anticancer applications [7,8,9]. Recent investigations have demonstrated the anticancer mechanisms of cepharanthine, which include the inhibition of cancer cell growth, the activation of programmed cell death, the disruption of cell division cycles, and the inhibition of metastasis [10,11,12]. Given that unlimited proliferative capacity represents a fundamental characteristic of malignant cells, inhibiting tumor cell proliferation serves as a crucial strategy in impeding cancer progression and plays an integral role in the overall antitumor response [13].

Research has revealed that cepharanthine has multiple antiproliferative effects on various cancer types. In hepatocellular carcinoma, it downregulates c-Myc expression and modulates amino acid and cholesterol metabolism, effectively suppressing tumor growth [14]. In oral squamous cell carcinoma, it reduces Ki67 expression, indicating decreased cellular proliferation, which is correlated with diminished FOXL2 transcription factor expression in the pterygoid spiral region [15]. This compound effectively inhibits the calcium-activated chloride channel Anoctamin-1 through the suppression of oxidized ANO1 currents and has significant antiproliferative effects on lung adenocarcinoma cells both in vitro and in vivo [10]. In cervical cancer, cepharanthine suppresses p38 MAPK and ERK1/2 phosphorylation within the MAPK/ERK signaling pathway by downregulating methionine adenosyltransferase 2B gene expression, resulting in proliferation inhibition and notable antitumor activity [15]. Additionally, it modulates reactive oxygen species (ROS) generation and mitochondrial pathways, contributing to apoptotic effects across various cancer cell types [16,17]. Elevated ROS levels can induce cellular damage through the oxidation of DNA, proteins, and lipids, ultimately triggering tumor cell apoptosis or necrosis [18]. This process facilitates cytochrome c and ROS release, activating caspase-9 and caspase-3, thereby initiating mitochondrial apoptosis. Cepharanthine-induced ROS accumulation activates c-Jun N-terminal kinase (JNK), further promoting apoptosis through the indirect regulation of Bcl-2 family proteins [19]. In glioblastoma multiforme cell lines, cepharanthine narrows voltage-dependent anion channels between the mitochondria and cytoplasm, promoting ROS efflux from the mitochondria to the cytoplasm and subsequently triggering apoptotic cell death [20].

Studies have also demonstrated that cepharanthine interacts with the Keap1-Nrf2/HO-1 pathway, a crucial cellular defense mechanism against oxidative stress and inflammation. In LPS-stimulated macrophages and murine colitis models, cepharanthine significantly increased Nrf2 expression and nuclear translocation, which in turn upregulated its downstream antioxidant targets HO-1 and NQO1. This activation depends on upstream AMPK-α1/AKT/GSK-3β signaling, and notably, cepharanthine failed to confer protective effects in Nrf2 knockout mice [21]. Furthermore, in gastric cancer, cepharanthine suppressed Keap1 expression and enhanced Nrf2 nuclear translocation, significantly increasing the transcription of key antioxidant genes such as NQO1, HO-1, and glutamate–cysteine ligase modifier subunit (GCLM) while concurrently increasing oxidative stress and disrupting mitochondrial function [22]. Together, these studies indicate that cepharanthine affects the Keap1-Nrf2/HO-1 axis to amplify cellular antioxidative defenses and anticancer activities. With respect to cell cycle regulation, cepharanthine enhances p21^Waf1^ expression in ovarian cancer and p53-mutant colorectal cancer cells while suppressing cyclin A and cyclin D levels, inducing G1 phase cell cycle arrest, and effectively inhibiting ovarian cancer cell proliferation [23,24].

On the basis of the current therapeutic landscape, treatment for recurrent or advanced cervical cancer remains suboptimal, with limited efficacy and poor survival outcomes. Cepharanthine has demonstrated anticancer properties across various cancer types and has been shown to have inhibitory effects on key pathways, including the MAPK/ERK and oxidative stress-related signaling pathways. In addition, its capacity to induce cell cycle arrest and modulate the Nrf2/HO-1 axis suggests its potential to target both proliferative and stress-adaptive mechanisms in cancer cells. These findings support the rationale for further investigating cepharanthine as a candidate for cervical cancer treatment. However, the detailed mechanisms remain poorly understood, especially in patients with cervical cancer.

## 2. Materials and Methods

### 2.1. Cell Culture

Three human cervical cancer cell lines, CaSki (#60251), HeLa (#60005), and C33A (#60554), were procured from the Bioresource Collection and Research Center (BCRC; Hsinchu, Taiwan; derived from ATCC^®^ CRM-1550^TM^, ATCC^®^ CRM-CCL-2^TM^, and ATCC^®^ CRM-HTB-31^TM^). CaSki cells were cultured in RPMI 1640 medium (SH30011.02, Cytiva, Marlborough, MA, USA) supplemented with 10% fetal bovine serum (FBS, SH30396.03, Cytiva), whereas HeLa and C33A cells were cultured in Eagle’s minimum essential medium (SH30024.02, Cytiva) supplemented with 10% FBS. The cells were maintained at 37 °C in a humidified incubator containing 5% CO_2_. Cepharanthine (CAS481-49-2, >95% purity, Abcam, Cambridge, UK) was solubilized in DMSO (D26650; Sigma-Aldrich, St. Louis, MO, USA) to generate stock solutions, and the final DMSO concentration in the cell culture was 0.1%. The cell lines were exposed to various concentrations of cepharanthine (0, 25, or 50 µM) for either 24 h or 48 h. Control group cells were treated with the same concentration of DMSO as the cepharanthine groups (0.1%). Following treatment, the cells were harvested for subsequent analysis.

### 2.2. Cell Viability Assay

Cells were plated in round-bottom clear 96-well plates (1 × 10^4^ cells/well) in 100 μL medium. After 24 h, the cells were exposed to cepharanthine (0, 25 or 50 µM) for 24 h or 48 h. After treatment, 100 μL of CCK-8 solution (CK04, Dojindo Laboratories, Rockville, MD, USA) was added per the manufacturer’s protocol. Absorbance measurements at 450 nm were performed using a microplate reader (Model: FLUOstar Galaxy; BMG Labtech, Ortenberg, Germany).

### 2.3. Colony Formation Assay

Cells were seeded in 6-well plates (1 × 10^3^ cells/well) and subjected to cepharanthine treatment (0, 25 or 50 µM) for 24 h. The culture medium was refreshed at 3-day intervals. Following a 14-day incubation period, the cells were washed with PBS, fixed with 4% formaldehyde for half an hour, and stained with 0.1% crystal violet solution for 30 min. Colony visualization was performed using a Scanner with a super high resolution 4800 × 4800 dpi (Epson Perfection V39, J371A, Seiko Epson Corporation, Nagano, Japan), and we used the thresholded binary (black and white with pixel intensity values between 132 and 255) for further analysis and colony quantification, conducted using ImageJ software (version 1.54c, https://imagej.net/ij/, accessed on 30 April 2024).

### 2.4. DAPI Staining Assay

Cells were seeded in 24-well plates (5 × 10^4^ cells/well) and exposed to cepharanthine (0, 25, or 50 µM) for 24 h. The cells were fixed with 4% formaldehyde for 30 min, followed by permeabilization with 0.1% Triton X-100 for 10 min. The cells were subsequently washed with PBS buffer and incubated with DAPI fluorescence dye (5 μM) for 30 min in the dark. Cellular morphological alterations were captured using a fluorescence microscope (Olympus DP73, Olympus Corporation, Shinjuku, Tokyo, Japan). Three fields per well at 400× magnification were documented, and we used the thresholded binary (black and white with pixel intensity values between 41 and 255) for analysis and quantification for each cell sample using ImageJ software.

### 2.5. Apoptosis and Cell Cycle Assays

The cells were seeded in 6-well plates (1 × 10^6^ cells/well). Following treatment with cepharanthine (0, 25, or 50 µM) for 24 h, the cells were harvested. Apoptosis was quantified using an FITC Annexin V apoptosis detection kit (#556547, BD Biosciences, Franklin, NJ, USA). Apoptotic analysis was conducted using an FC500 flow cytometer and CXP software (version 2.3; Beckman Coulter, Brea, CA, USA), and both early and late apoptotic/necrotic levels were measured. In further experiments, cells were treated simultaneously with ROS inhibitor, such as N-acetyl-L-cysteine (NAC, CAS616-91-1, >95% purity, Sigma-Aldrich, St. Louis, MO, USA), prior to cepharanthine treatment.

For cell cycle analysis, harvested cells were fixed in 70% ethanol at −20 °C overnight. After washing, the cells were resuspended in 500 μL of propidium iodide/RNase staining buffer (PI, 10 mg/mL; RNases, 300 mg/mL; BD Pharmingen, Franklin, NJ, USA). Following 15 min of incubation in the dark at ambient temperature, cell cycle distribution data were acquired with a flow cytometer, as described above.

### 2.6. Wound Healing Assay

Cells were seeded in 6-well plates (2 × 10^6^ cells/well). After 24 h, straight wounds were created on the monolayer cell surface using a 200 μL pipette tip. Following dual washing to eliminate cell debris, the cultures were subjected to cepharanthine treatment (0, 25, or 50 µM) for 24 h. Wound gaps were documented at 0 h and 24 h using an Olympus DP73 microscope, and we use the thresholded binary (black and white with pixel intensity values between 59 and 255) for further analysis. To increase the credibility of the experimental results, image collection was performed to capture multiple fields of view at different locations on a single wound area under each experimental condition, and the wound areas were quantified using ImageJ software. To minimize the result variability, a minimum of four observations per well were recorded at 40× magnification, and wound closure was assessed as a percentage relative to that of the untreated control.

### 2.7. Mitochondrial Membrane Potential (Δψm) Assay

The cells were seeded in 6-well plates (1 × 10^6^ cells/well) and subjected to cepharanthine treatment (0, 25, or 50 µM) for 24 h. The mitochondrial membrane potential was evaluated using the BD MitoScreen Kit (#551302, BD Biosciences). The samples were analyzed using a flow cytometer as described above. Fluorescence emission was detected at 527 nm for the monomeric form and 590 nm for the J-aggregates.

### 2.8. ROS Assay

Cells were seeded in 6-well plates (1 × 10^6^ cells/well) and treated with cepharanthine (0, 25, or 50 µM) for 24 h. Following centrifugation (400× *g* for 5 min) and supernatant removal, the cells were resuspended in 500 μL of H2DCFDA fluorescein dye (#4091-99-0, 10 µmol/L, 2′,7′-dichlorodihydrofluorescein diacetate, Sigma-Aldrich) and incubated for 20 min at 37 °C in the dark. The fluorescence signals were subsequently analyzed using FC500 flow cytometry and FlowJo software (version 7.6; BD Life Sciences, Franklin Lakes, NJ, USA). In further experiments, cells were treated simultaneously with NAC (Sigma-Aldrich), prior to cepharanthine treatment.

### 2.9. Total Superoxide Dismutase (T-SOD) and Total Glutathione (T-GSH)/Oxidized Glutathione (GSSG) Assays

Cells were cultured in 10 cm dishes (2 × 10^6^ cells/well) and exposed to cepharanthine (0, 25, or 50 µM) for 24 h. The total superoxide dismutase (T-SOD) activity was analyzed using a T-SOD Activity Assay Kit (E-BC-K020-M, Elabscience, Houston, TX, USA), utilizing WST-1. The absorbance was measured at 450 nm using a CLARIOstar fluorescence microplate reader (BMG Labtech). The protein concentration of the supernatant was determined using a BCA protein assay kit (#23225, Thermo Fisher, Waltham, MA, USA). For T-GSH and GSSG quantification, a total glutathione (T-GSH)/oxidized glutathione (GSSG) colorimetric assay kit (E-BC-K097-M, Elabscience) with DNTB was used. Sample preparation followed the manufacturer’s protocols, and the T-GSH and GSSG levels were measured at 412 nm using a CLARIOstar fluorescence microplate reader (BMG Labtech).

### 2.10. Western Blotting

Cells were seeded into 10 cm culture dishes (2 × 10^6^ cells/well) and subjected to cepharanthine treatment (0, 25, or 50 µM) for 24 h. The proteins were extracted using RIPA buffer (#20-188, Millipore, Billerica, MA, USA), and the protein concentrations were determined utilizing a BCA protein assay kit (#23225, Thermo Fisher). Cell lysate protein (30 μg) was separated via 10–12% (*w*/*v*) SDS–PAGE followed by transfer onto PVDF membranes (0.2 mm; EA162-0177, Bio-Rad, Irvine, CA, USA). The membranes were blocked with BlockPRO protein-free blocking buffer (#BF01-1L, Energenesis Biomedical, Taipei, Taiwan) for 1 h before being incubated overnight at 4 °C with primary antibodies (1:1000), which included the following primary antibodies: PARP (46D11) (#9532), Bcl-2 (D55G8) (#15071), Bax (D2E11) (#5023), cleaved caspase-3 (Asp175) (#9664), phospho-AMPKα (Thr172) (#2535), AMPKα (D63G4) (#5832), phospho-p53 (S15) (#9284), p53 (#9282), Nrf2 (D1Z9C) (#12721), Keap1 (D6B12) (#8047), NQO1 (A180) (#3187), HO-1 (E3F4S) (#43966) (all from Cell Signaling Technology, Danvers, MA, USA), and GAPDH (MA5-15738; Thermo Fisher). Following washing, the membranes were incubated for 1 h with the following secondary antibodies: HRP-conjugated goat anti-mouse IgG (#115-035-003, 1:50,000) or anti-rabbit IgG (#111-035-003, 1:100,000; Jackson ImmunoResearch Laboratories, West Grove, PA, USA). Blot detection was performed with Clarity and Clarity Max ECL Western Blotting Substrates (#1705062, Bio-Rad), and blot intensity analysis was carried out using FusionCapt Advance FX7 software (version 16.08a; Vilber Lourmat, Collégien, France).

### 2.11. Tumor Xenograft Mouse Experiments

Twenty-four female BALB/c mice (7 weeks old) were obtained from the National Laboratory Animal Center (Taipei, Taiwan). The animals were maintained in a pathogen-free facility with controlled environmental conditions (22 °C, 12:12 h light/dark cycle). The experimental protocol received approval from the Institutional Animal Care and Use Committee (IACUC) of Changhua Christian Hospital, Taiwan (approval no: CCH-AE-112-008, accessed on 31 July 2023). Two parts of human cervical cancer C33A cells (1 × 10^7^) were mixed with one part of Matrigel (#354248; Corning, Tewksbury, MA, USA), and 200 μL of this mixture was subcutaneously injected into the right flank of each animal. After the tumor volume reached approximately 250 mm^3^ (day 7), the mice tumor volumes were averagely assigned to three groups (*n* = 8/group). The groups received intraperitoneal injections (i.p.) of either vehicle control [150 μL, comprising 10% DMSO, 40% Cremophor/ethanol (C5135, Sigma-Aldrich), in PBS] or cepharanthine [15 or 30 mg/kg, dissolved in identical vehicle solution] every other day for 16 days (until Day 23). The cepharanthine doses used in this study were chosen on the basis of the previously published experiments [22,25]. Throughout the study, body weights and tumor dimensions were monitored using an electronic caliper. The tumor volumes were calculated according to the following formula: (length × width^2^)/2. Tumor volumes also were allowed to grow until the human endpoint criteria exceeded 2000 mm^3^, or the mice appeared with an ulcerated tumor or showed sick or moribund status, at which point the mice were euthanized. Otherwise, the mice were euthanized on day 25 in a CO_2_ chamber, followed by tumor excision and weight measurement for each group.

### 2.12. Histopathology and Immunohistochemistry Analyses

The excised tumor samples were preserved in 10% neutral buffered formalin (Leica Biosystems, Richmond, IL, USA) followed by paraffin embedding. Tissue sections (5 μm thick) were prepared and subjected to immunohistochemical (IHC) analysis. After deparaffinization and rehydration, epitope retrieval was performed by using citrate buffer HIER solution (CBB500, ScyTek Laboratories, Logan, UT, USA). After blocking with Novolink Polymer Detection Systems (RE7158, Leica Biosystems), the sections were incubated with primary antibodies, including ki67 (#12202; 1:200), cleaved caspase-3 (Asp175) (#9664; 1:100), phospho-AMPKα (Thr172) (#2535; 1:100), phospho-p53 (S15) (#9284; 1:50), and Nrf2 (D1Z9C) (#12721; 1:200) (all from Cell Signaling Technology), overnight at 4 °C. After being washed, the sections were incubated with a rabbit antibody enhancer and a polymer-HRP rabbit (D39-18, Rockville, MD, USA) for 15 min each. Visualization was achieved using 3,3′-diaminobenzidine (DAB) chromogen solution (RE7270-K, Leica Microsystems, Wetzlar, Germany) for 5 min, followed by hematoxylin counterstaining. Images of the samples were acquired using an Olympus DP73 microscope system and using the IHC Toolbox plugin in ImageJ software; color thresholding was then adjusted with an intensity cutoff ≈ 150 ± 15 to isolate the DAB-positive regions. Image analysis and quantification were conducted on three fields per each specimen at 400× magnification utilizing ImageJ software.

### 2.13. Statistical Analysis

Data analysis was conducted using one-way analysis of variance (ANOVA) with Tukey’s multiple comparisons test as a post hoc analysis, utilizing GraphPad Prism software (version 9.3.0; Dotmatics, Boston, MA, USA). The results represent three independent experiments and are expressed as the mean ± standard deviation (SD), with statistical significance set at *p* < 0.05.

## 3. Results

### 3.1. Cepharanthine Inhibits Cervical Cancer Cell Viability and Colony Formation

**Figure 1:**
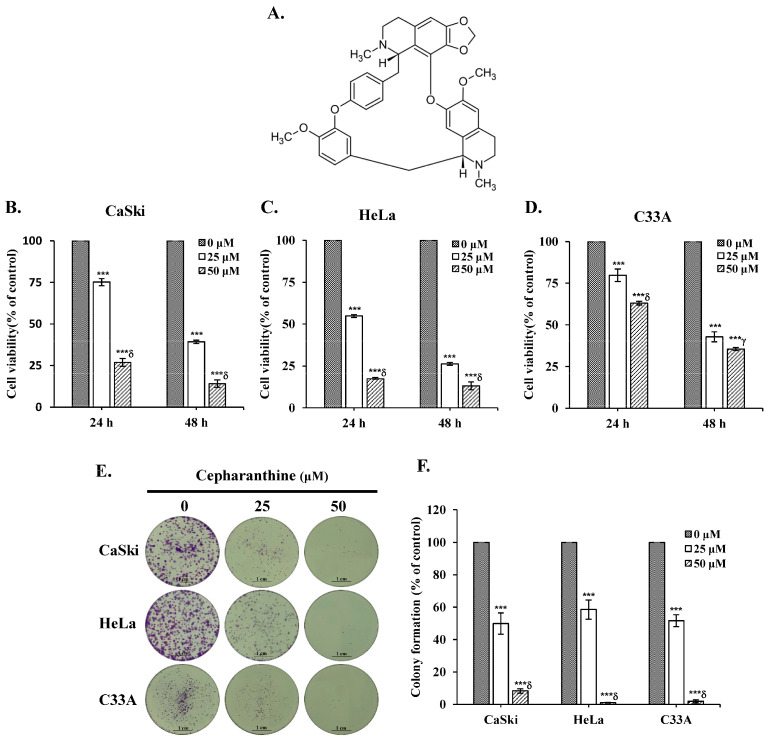
Cepharanthine reduces cell viability and colony formation in cervical cancer cell lines. (**A**) The structure of cepharanthine. The human cervical cancer cell lines CaSki (**B**), HeLa (**C**), and C33A (**D**) were treated with cepharanthine (0, 25, and 50 μM) for 24 or 48 h, and cell viability was measured using a CCK-8 assay. (**E**,**F**) CaSki, HeLa, and C33A cell lines were treated with cepharanthine (0, 25, and 50 μM) for 24 h, and the degree of colony formation was assessed by comparing the absorbance of the samples with crystal violet staining, scale bar = 1 cm. The values represent the means ± SDs from three replicates. *** *p* < 0.001 compared with the control (0.1% DMSO-treated cells); ^γ^ *p* < 0.01 and ^δ^ *p* < 0.001 compared with the Cep 25 μM treatment.

To assess whether cepharanthine exerts cytotoxic effects on cervical cancer cells, human cervical cancer cell lines (CaSki, HeLa and C33A) were treated with different concentrations of cepharanthine (25 and 50 µM) for 24 and 48 h, and cell viability was assayed using the CCK-8 method. Figure 1A depicts the structure of cepharanthine. The results revealed that cepharanthine significantly suppressed CaSki, HeLa, and C33A cell viability in a dose- and time-dependent manner (Figure 1B–D, *p* < 0.05). The viability of the three cell lines after 24 h of treatment was 75.2%, 54.8%, and 79.8% with 25 µM cepharanthine and 26.9%, 17.4%, and 63.0% with 50 µM cepharanthine, respectively. However, after 48 h of treatment, it was 39.3%, 26.3%, and 42.8% with 25 µM cepharanthine and 14.1%, 13.2%, and 35.5% with 50 µM cepharanthine, respectively (Table S1). On the basis of these results, treatment with cepharanthine (25 and 50 µM) for 24 h was used to examine its effect on the subsequent cell experiments in this study. Similar results were obtained with crystal violet staining; treatment with 25 or 50 µM cepharanthine significantly inhibited colony formation by 50.1% and 91.7% in CaSki cells, by 41.5% and 98.9% in HeLa cells, and by 48.3% and 98.1% in C33A cells, compared with those in the control group (Figure 1E,F and Table S1, *p* < 0.05). These results clearly revealed that cepharanthine has antiproliferative effects on cervical cancer cells.

### 3.2. Cepharanthine Induces Apoptosis and Cell Cycle Arrest in Cervical Cancer Cells

**Figure 2:**
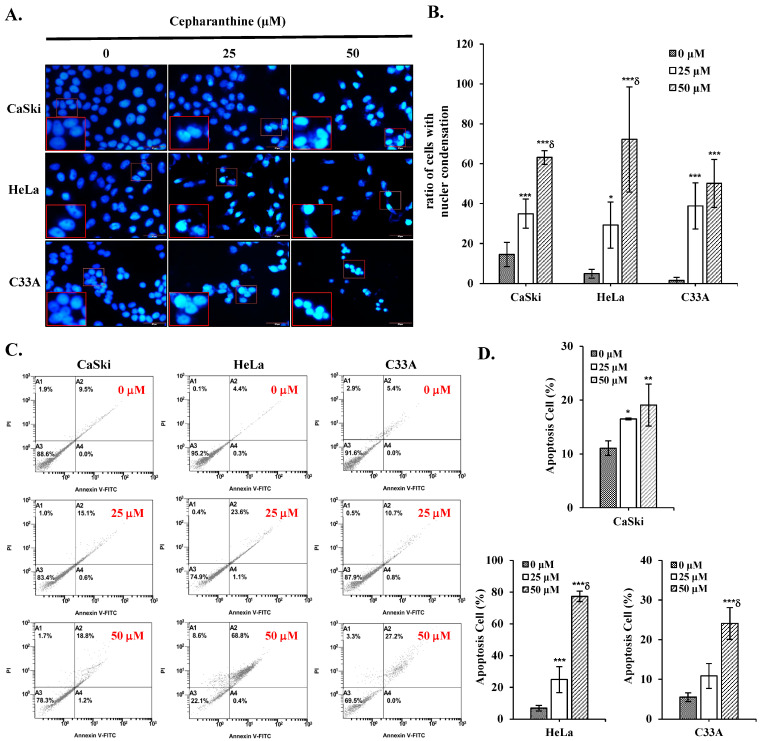
Cepharanthine triggers apoptotic cell death in cervical cancer cell lines. (**A**,**B**) Nuclear condensation evaluation through DAPI staining. CaSki, HeLa, and C33A cells were subjected to cepharanthine treatment (0, 25, and 50 μM) for 24 h, and fluorescence microscopy images were captured at 400× magnification; scale bar = 50 μm. Quantitative analysis of the relative density ratio of cells exhibiting nuclear condensation. (**C**,**D**) Apoptosis detection via Annexin V/PI staining with flow cytometric analysis. The relative density percentage of apoptotic cells (encompassing both early and late stages) was quantified. The values represent the means ± SDs from three replicates. * *p* < 0.05, ** *p* < 0.01, and *** *p* < 0.001 compared with the control (0.1% DMSO-treated cells); ^δ^ *p* < 0.001 compared with the Cep 25 μM treatment.

**Figure 3:**
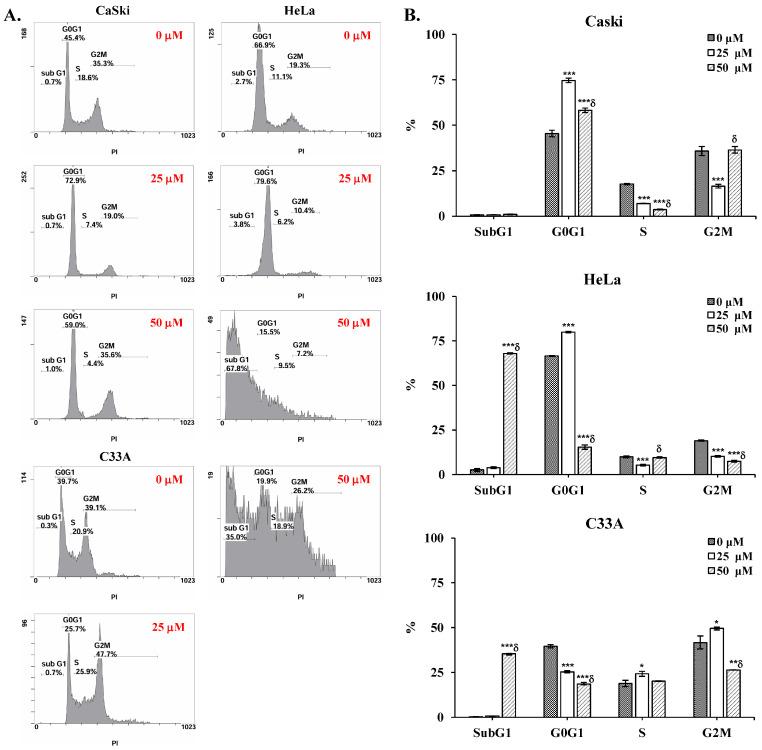
Cepharanthine induces cell cycle arrest at the sub-G1 and G0/G1 phases in cervical cancer cell lines. (**A**) Cell cycle distribution analysis through PI staining. CaSki, HeLa, and C33A cells were exposed to cepharanthine (0, 25, and 50 μM) for 24 h, followed by flow cytometric analysis. (**B**) Quantitative assessment of relative density percentages across cell cycle phases (sub-G1, G0/G1, S, and G2/M). The values are presented as the means ± SDs from triplicate experiments. * *p* < 0.05, ** *p* < 0.01, and *** *p* < 0.001 compared with the control (0.1% DMSO-treated cells); ^δ^ *p* < 0.001 compared with the Cep 25 μM treatment.

To determine whether cepharanthine can induce apoptotic death and cell cycle arrest in cervical cancer cells. CaSki, HeLa, and C33A cells were exposed to 25 and 50 µM cepharanthine for 24 h and stained with Annexin V/PI and PI/RNase for flow cytometry analysis. Furthermore, cepharanthine-treated CaSki, HeLa, and C33A cells were also stained with DAPI and observed via fluorescence microscopy. As shown in Figure 2A,B, compared with the vehicle-treated controls, cepharanthine treatment significantly increased nuclear fragmentation and nuclear condensation in a concentration-dependent manner (CaSki cells, 14.5 ± 6.0 vs. 35.0 ± 7.3 vs. 63.2 ± 3.5; HeLa cells, 4.9 ± 2.1 vs. 29.2 ± 11.6 vs. 72.2 ± 26.4; C33 A cells, 1.5 ± 1.7 vs. 38.9 ± 11.6 vs. 50.1 ± 12.1, Table S1, *p* < 0.05) and significantly inhibited cell growth. Moreover, there were significantly more apoptotic cells in the cepharanthine treatment group than in the vehicle-treated control group (CaSki cells, 11.1 ± 1.3% vs. 16.5 ± 0.2% vs. 19.1 ± 3.9%; HeLa cells, 6.8 ± 1.8% vs. 24.8 ± 8.1% vs. 77.3 ± 3.3%; C33 A cells, 5.5 ± 1.1% vs. 10.9 ± 3.1% vs. 24.1 ± 4.0%; Figure 2C,D and Table S1; *p* < 0.05). In the cell cycle distribution assay, the highest concentration of cepharanthine (50 µM) significantly increased the percentage of G0/G1-phase cells from 45.5 ± 1.8% to 58.2 ± 1.2% in CaSki cells, and the percentage of sub-G1-phase cells significantly increased from 2.6 ± 0.9% to 68.0 ± 0.3% and 0.3 ± 0.1% to 35.4 ± 0.3% in HeLa and C33A cells, respectively (Figure 3 and Table S1, *p* < 0.05). These results suggested that cepharanthine-mediated suppression of cervical cancer cell growth was partly due to apoptosis and sub-G1- and G0/G1-phase arrest.

### 3.3. Cepharanthine Inhibits Cervical Cancer Cell Migration

**Figure 4:**
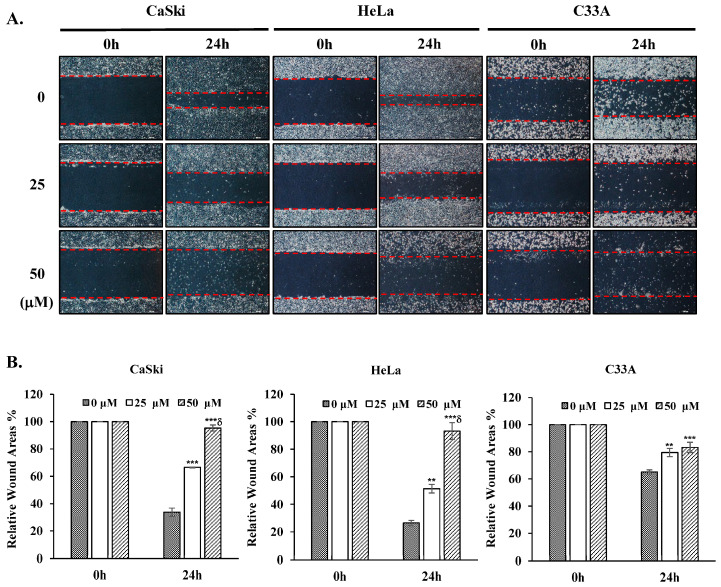
Cepharanthine suppresses cervical cancer cell migration. (**A**) CaSki, HeLa, and C33A cells were subjected to cepharanthine treatment (0, 25, and 50 μM) before wound creation using a 200 μL sterile pipette tip, and microscopy images were captured at 40× magnification; scale bar = 500 μm. The extent of wound closure was evaluated 24 h post-wounding. (**B**) Semi-quantitative analysis of relative wound areas through wound width measurement (red lines). The values are expressed as the means ± SDs from three independent replicates. ** *p* < 0.01 and *** *p* < 0.001 compared with the control (0.1% DMSO-treated cells); ^δ^ *p* < 0.001 compared with the Cep 25 μM treatment.

To examine the impact of cepharanthine on cervical cancer cell migration, we conducted a wound healing assessment. Our findings revealed a notable decrease in the migratory capacity of CaSki, HeLa, and C33A cells in the cepharanthine (25 and 50 µM) treatment groups compared with the vehicle-treated control groups at 24 h, by 33% and 62%, respectively, in CaSki cells, by 25% and 67%, respectively, in HeLa cells and by 14% and 18%, respectively, in C33A cells (Figure 4, *p* < 0.05). These results suggest that cepharanthine has antimetastatic effects on human cervical cancer cells.

### 3.4. Cepharanthine Depolarizes the Mitochondrial Membrane Potential (∆ψm) and Activates the AMPK/p53 Pathway to Mediate the Mitochondrial Apoptotic Pathway in Cervical Cancer Cells

**Figure 5:**
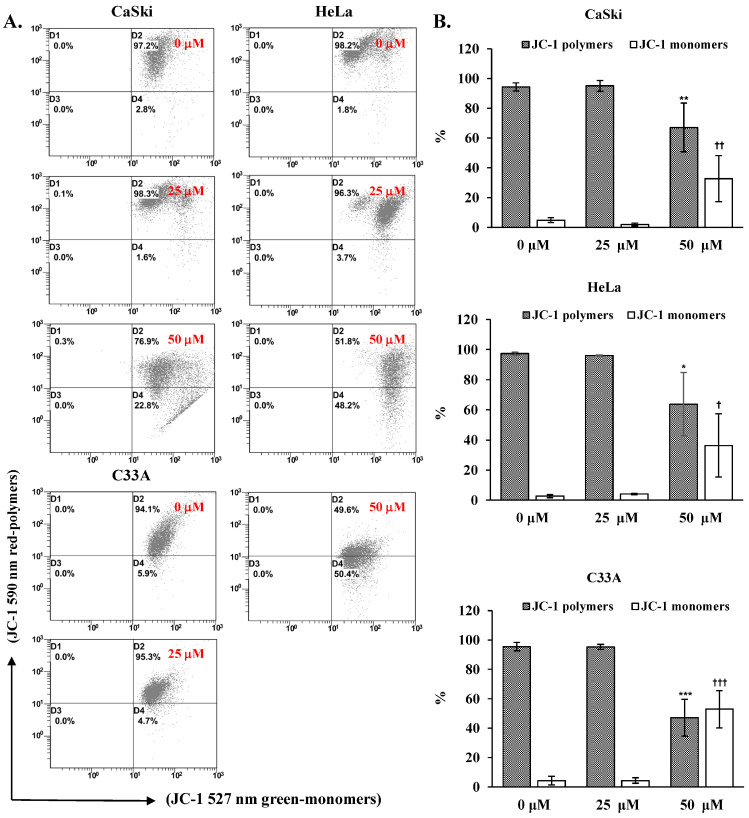
Cepharanthine induces mitochondrial membrane potential (∆ψm) depolarization in cervical cancer cell lines. (**A**) Assessment of ∆ψm via JC-1 staining methodology. CaSki, HeLa, and C33A cells were subjected to cepharanthine treatment (0, 25, and 50 μM) for 24 h, followed by flow cytometric analysis. (**B**) Quantitative evaluation of red-polymer (595 nm) and green-monomer (525 nm) fluorescence percentages. The data are presented as the means ± SDs from triplicate experiments. * *p* < 0.05, ** *p* < 0.01, and *** *p* < 0.001, comparing the control group with the polymer-treated group; ^†^ *p* < 0.05, ^††^ *p* < 0.01, and ^†††^ *p* < 0.001, comparing the control group with the monomer-treated group. Control, 0.1% DMSO-treated cells.

**Figure 6:**
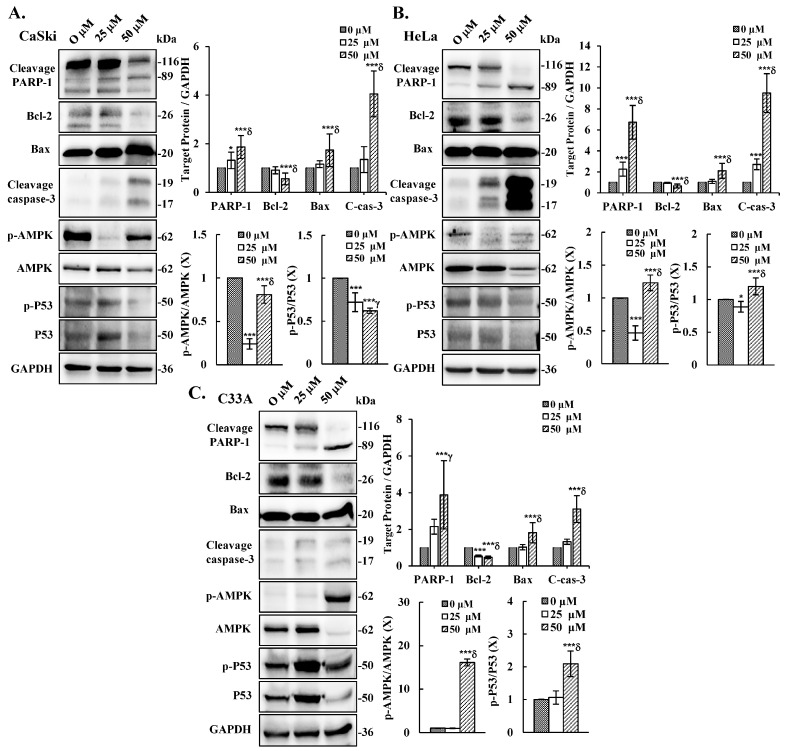
Cepharanthine induces apoptosis through the activation of apoptosis-associated proteins and AMPK/P53 phosphorylation in cervical cancer cells. CaSki (**A**), HeLa (**B**), and C33A (**C**) cells were subjected to cepharanthine treatment (0, 25, and 50 μM) for 24 h, followed by Western blot analysis of PARP-1, Bcl-2, Bax, cleaved caspase-3, total and phosphorylated AMPK, and P53 levels, with GAPDH as a loading control. The results of the quantitative analyses are presented in the plots on the right. The values represent the means ± SDs from three replicates. * *p* < 0.05 and *** *p* < 0.001 compared with the control (0.1% DMSO-treated cells); ^γ^ *p* < 0.01 and ^δ^ *p* < 0.001 compared with the Cep 25 μM treatment.

To elucidate the molecular mechanism underlying cepharanthine-mediated apoptosis, we exposed CaSki, HeLa, and C33A cells to cepharanthine concentrations of 25 and 50 µM for 24 h. As demonstrated in Figure 5, alterations in the mitochondrial membrane potential were observed, with a substantial increase in depolarized cells at 50 µM cepharanthine. The green fluorescence intensity increased by 28%, 34%, and 49% in CaSki, HeLa, and C33A cells, respectively, relative to that in the vehicle-treated controls (Table S1, *p* < 0.05), indicating impaired mitochondrial functionality. Western blotting analysis also revealed that the highest cepharanthine concentration significantly increased the expression of proapoptotic cleaved PARP-1, Bax, and cleaved caspase 3 and decreased the expression of antiapoptotic Bcl-2 proteins (CaSki cells, ratios to control: 1.87 ± 0.47 vs. 1.74 ± 0.67 vs. 4.05 ± 0.94 vs. 0.56 ± 0.24; HeLa cells, ratios to control: 6.73 ± 1.61 vs. 2.11 ± 0.71 vs. 9.51 ± 1.86 vs. 0.67 ± 0.19; C33A cells, ratios to control: 3.88 ± 1.86 vs. 1.82 ± 0.55 vs. 3.11 ± 0.73 vs. 0.47 ± 0.07, respectively; Figure 6 and Table S1, *p* < 0.05), compared with the vehicle-treated controls. Furthermore, the involvement of the AMPK/p53 pathway in apoptotic regulation in cepharanthine-induced apoptosis was also investigated via Western blotting. In CaSki cells, the highest level of cepharanthine significantly decreased the phosphorylation of AMPK and p53 (ratios to control: 0.81 ± 0.10 vs. 0.62 ± 0.03). In contrast, the phosphorylation of AMPK and p53 was significantly higher in both HeLa and C33A cells than in the vehicle-treated controls (HeLa cells, ratios to control: 1.23 ± 0.12 vs. 1.20 ± 0.13; C33A cells, ratios to control: 16.1 ± 0.78 vs. 2.09 ± 0.39, respectively, *p* < 0.05). These results clearly suggest that cepharanthine causes mitochondrial membrane potential depolarization, activates the mitochondria-mediated apoptotic pathway, and then correlates with the AMPK/p53 pathway, causing the death of cervical cancer cells.

### 3.5. Cepharanthine Treatment Induces Oxidative Stress in Cervical Cancer

**Figure 7:**
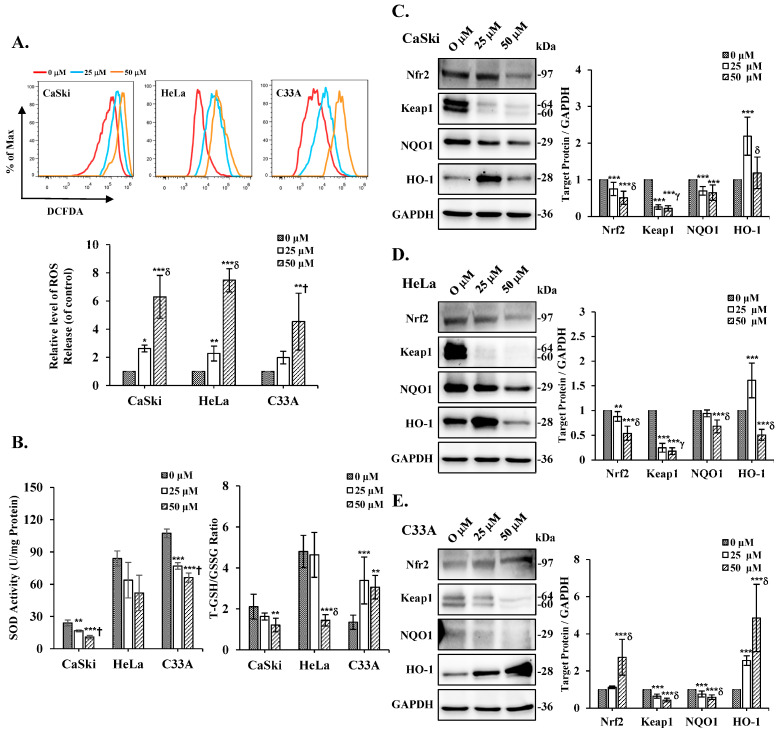
Effects of cepharanthine administration on oxidative stress markers in cervical cancer cell lines. CaSki, HeLa, and C33A cells were subjected to cepharanthine treatment (0, 25, and 50 μM) for 24 h. (**A**) Intracellular ROS levels were evaluated through flow cytometric analysis utilizing DCFDA staining. (**B**) ELISA was used to determine the SOD content and the GSH/GSSG ratio. (**C**–**E**) Western blot analysis was performed to assess the protein expression levels of Nrf2, Keap1, NQO1, and HO-1, with GAPDH as the internal reference. The quantitative analysis results are presented in the plots on the right. The values are expressed as the means ± SDs from three independent experiments. * *p* < 0.05, ** *p* < 0.01, and *** *p* < 0.001 compared with the control (0.1% DMSO-treated cells); ^†^ *p* < 0.05, ^γ^ *p* < 0.01, and ^δ^ *p* < 0.001 compared with the Cep 25 μM treatment.

On the basis of the previous results, cepharanthine evidently causes the loss of the mitochondrial membrane potential. Therefore, to investigate whether oxidative stress is involved in cell cepharanthine toxicity, the amount of ROS in the cells was examined using DCFDA staining via flow cytometry analysis. We observed that the intracellular ROS levels were significantly increased following treatment with cepharanthine (25 and 50 µM) in CaSki, HeLa, and C33A cells, compared with those in the vehicle-treated controls (CaSki cells, ratios to control: 2.63 ± 0.24 vs. 6.30 ± 1.52; HeLa cells, ratios to control: 2.27 ± 0.53 vs. 7.47 ± 0.82; C33A cells, ratios to control: 1.98 ± 0.45 vs. 4.53 ± 2.02, respectively, Figure 7A and Table S1, *p* < 0.05). Next, we used ELISA to assess the SOD, GSH, and GSSG expression levels to understand how oxidative stress is induced. The results revealed that the CaSki, HeLa, and C33A cells had lower levels of SOD activity than did the control cells exposed to cepharanthine (CaSki cells, 24.0 ± 2.7 U/mg vs. 16.7 ± 0.8 U/mg vs. 10.9 ± 1.6 U/mg; HeLa cells, 84.0 ± 7.1 U/mg vs. 63.9 ± 16.5 U/mg vs. 51.9 ± 16.4 U/mg; C33A cells, 107.4 ± 4.1 U/mg vs. 77.0 ± 3.0 U/mg vs. 66.1 ± 4.3 U/mg, respectively. Similar results were observed, whereby cepharanthine treatment also significantly decreased the GSH/GSSG ratio with higher oxidative stress in CaSki and HeLa cells, although in C33A cells, it was increased (CaSki cells, 2.11± 0.60 vs. 1.63 ± 0.17 vs. 1.21 ± 0.33; HeLa cells, 4.80 ± 0.79 vs. 4.64 ± 1.10 vs. 1.44 ± 0.28; C33A cells, 1.35 ± 0.35 vs. 3.39 ± 1.15 vs. 3.06 ± 0.57 U/mg, respectively, Figure 7B and Table S1, *p* < 0.05). These findings indicate that oxidative stress occurs in cells receiving cepharanthine therapy and that oxidative stress levels increase during cepharanthine-induced apoptotic cell death.

### 3.6. Cepharanthine Induced Apoptosis by Regulating the Nrf2/Keap1 Pathway to Exert Oxidative Stress in Cervical Cancer Cells

To determine the role of the Nrf2/Keap1 signaling cascade in cepharanthine-induced oxidative stress, we analyzed the protein expression patterns of Nrf2, Keap1, NQO1, and HO-1 in cepharanthine-treated cervical cancer cells using Western blot analysis. As shown in Figure 7C, substantial reductions in Nrf2, Keap1, and NQO1 protein levels were detected in CaSki cells treated with 50 μM cepharanthine compared with those in the vehicle-treated controls (ratios to control: 0.51 ± 0.18 vs. 0.22 ± 0.07 vs. 0.65 ± 0.21, respectively, *p* < 0.05), whereas HO-1 protein expression was elevated relative to that in the controls (ratios to control: 1.19 ± 0.43, Table S1, *p* < 0.05). Similar results were observed for HeLa cells, in which the highest cepharanthine treatment also significantly decreased Nrf2, Keap1, NQO1, and HO-1 protein levels (ratios to control: 0.54 ± 0.14 vs. 0.18 ± 0.07 vs. 0.68 ± 0.13 vs. 0.51 ± 0.11, respectively; Figure 7D and Table S1, *p* < 0.05). In C33A cells, Keap1 and NQO1 were significantly decreased after the highest cepharanthine treatment, whereas Nrf2 and HO-1 were significantly increased compared with those in the vehicle-treated controls (ratios to control: 0.43 ± 0.10 vs. 0.59 ± 0.12 vs. 2.74 ± 0.96 vs. 4.85 ± 1.81, respectively, Figure 7E and Table S1, *p* < 0.05). Taken together, these findings suggest that cepharanthine-induced apoptosis affects oxidative stress and regulates the Nrf2/Keap1 axis.

### 3.7. NAC Inhibits ROS Levels and Reverses Cepharanthine-Induced Apoptosis in Cervical Cancer Cells

**Figure 8:**
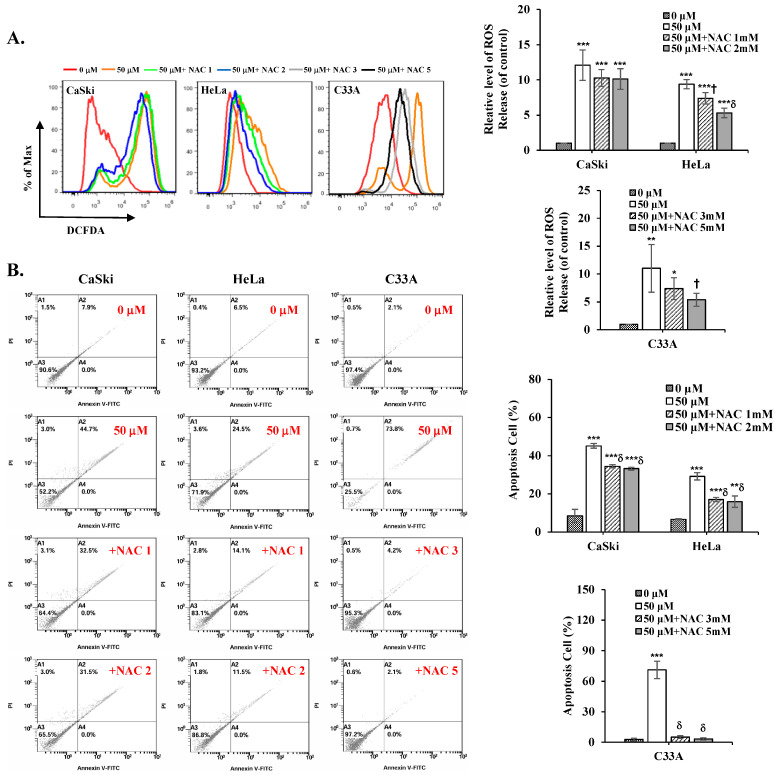
NAC inhibits ROS levels and reverses cepharanthine-induced apoptosis in cervical cancer cell lines. CaSki, HeLa, and C33A cells were subjected to cepharanthine treatment (0 and 50 μM) with or without NAC (1, 2, 3 and 5 mM) for 24 h. (**A**) Intracellular ROS levels were evaluated through flow cytometric analysis utilizing DCFDA staining. (**B**) Apoptosis detection via Annexin V/PI staining with flow cytometric analysis. The relative density percentage of apoptotic cells (encompassing both early and late stages) was quantified. The values are expressed as the means ± SDs from three independent experiments. * *p* < 0.05, ** *p* < 0.01, and *** *p* < 0.001 compared with the control (0.1% DMSO-treated cells); ^†^ *p* < 0.05 and ^δ^ *p* < 0.001 compared with the Cep 50 μM treatment.

To further validate the role of ROS in cepharanthine-induced apoptosis, we used the ROS scavenger NAC in further experiments. As shown in Figure 8A, NAC significantly inhibited the intracellular ROS levels, which were increased in the treatment with cepharanthine (CaSki cells, ratios to control: 12.11 ± 2.16 vs. 10.28 ± 1.19 vs. 10.11 ± 1.47; HeLa cells, ratios to control: 9.39 ± 0.66 vs. 7.39 ± 0.81 vs. 5.31 ± 0.67; C33A cells, ratios to control: 11.01 ± 4.25 vs. 8.51 ± 0.59 vs. 4.33 ± 1.36, respectively, Table S1, *p* < 0.05). Similar results were obtained for cepharanthine-induced apoptosis; NCA also significantly inhibited the apoptotic cells in the cepharanthine group (CaSki cells, 8.37 ± 3.39 vs. 45.15 ± 1.20 vs. 34.43 ± 0.75 vs. 33.20 ± 0.80; HeLa cells, 6.55 ± 0.18 vs. 29.10 ± 1.94 vs. 16.98 ± 1.13 vs. 15.77 ± 3.00; C33A cells, 2.62 ± 1.58 vs. 71.08 ± 8.55 vs. 5.08 ± 1.37 vs. 3.30 ± 1.313, respectively, Figure 8B and Table S1, *p* < 0.05). These results clearly indicated that the addition of ROS levels might be the reason for cepharanthine-induced apoptosis.

### 3.8. Cepharanthine Suppresses Tumor Development in Cervical Cancer Cell-Derived Xenograft Nude Mice

**Figure 9:**
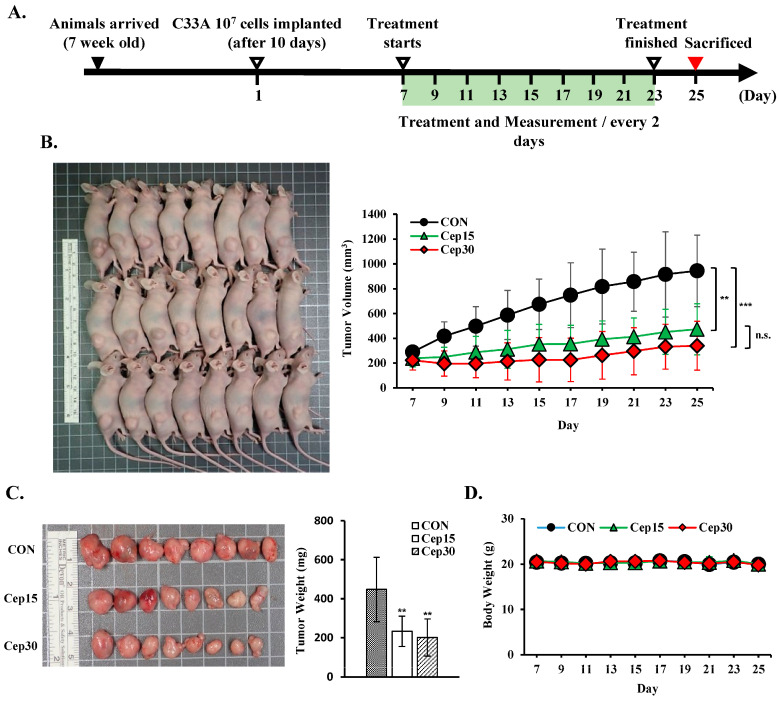
Cepharanthine inhibits tumor progression in a cervical cancer xenograft model. C33A cells (1 × 10^7^ cells) were subcutaneously implanted into the right flank region of BALB/c nude mice. Following a 7-day period, the intraperitoneal administration of cepharanthine (15 mg/kg or 30 mg/kg) was initiated every other day. (**A**) Experimental design schematic. (**B**) Representative images of tumors from C33A xenograft nude mice and measurement data of tumor volume (**C**) were obtained on day 25. (**D**) Body weight measurements were recorded every two days. The values represent the means ± SDs (*n* = 8/group). ** *p* < 0.01, *** *p* < 0.001 compared with the CON group; n.s. (not statistically significant) compared with the Cep15-treated group. CON, control; Cep15, 15 mg/kg cepharanthine; Cep30, 30 mg/kg cepharanthine.

**Figure 10:**
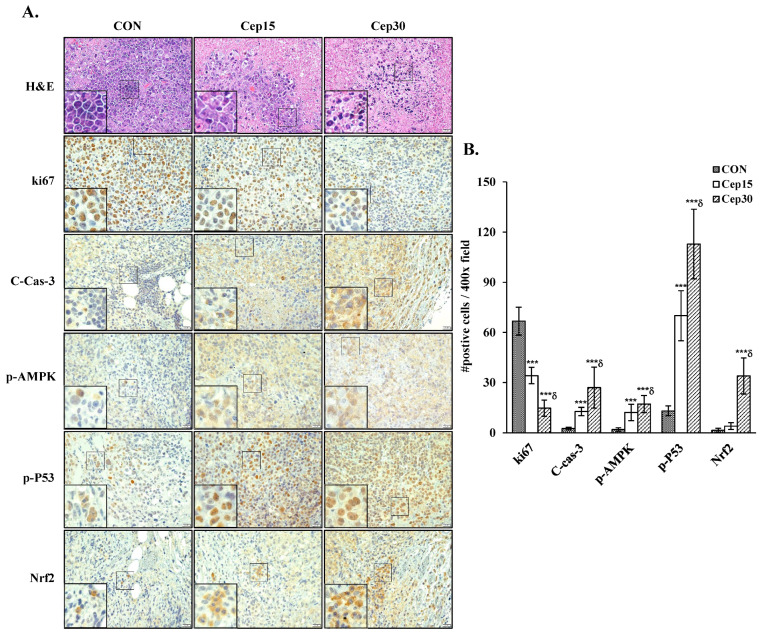
Cepharanthine decreases the protein expression of ki67, cleaved caspase3, p-AMPK, p-P53, and Nrf2 in C33A xenograft nude mice. (**A**) Representative histological sections of tumor samples subjected to hematoxylin and eosin, ki67, cleaved caspase-3, p-AMPK, p-P53, and Nrf2 staining (brown coloration; 400× magnification, scale bar = 20 μm). (**B**) The results of the quantitative analyses are presented in the plot on the right. The values are expressed as the means ± SDs (*n* = 8/group), which was conducted on three fields per specimen at 400× magnification utilizing ImageJ software. *** *p* < 0.001 compared with the control; ^δ^ *p* < 0.001 compared with the Cep 25 μM treatment. CON, control; Cep15, 15 mg/kg cepharanthine; Cep30, 30 mg/kg cepharanthine.

To assess the effect of cepharanthine on cervical cancer cell growth in vivo, we subcutaneously implanted C33A cells into BALB/c nude mice. The experimental timeline is shown in Figure 9A. Following 18 days of treatment, cepharanthine significantly inhibited tumor growth, with C33A xenograft tumor volumes averaging 944.5 ± 289.2 mm^3^ vs. 473.3 ± 206.3 mm^3^ vs. 339.2 ± 197.6 mm^3^ in the vehicle-treated control group and cepharanthine (15 mg/kg)- and (30 mg/kg)-treated groups, resulting in tumor growth inhibition rates of 49.9% and 64.1%, respectively (Figure 9B and Table S2, *p* < 0.05). The average tumor weights were significantly lower in the cepharanthine (15 mg/kg)- and cepharanthine (30 mg/kg)-treated groups (232.8 ± 77.5 mg vs. 200.4 ± 95.1 mg, respectively) than in the vehicle-treated control group (448.1 ± 165.9 mg, Figure 9C and Table S2, *p* < 0.05), whereas the average body weights remained equal among the three groups (20.0 ± 0.6 g vs. 19.8 ± 1.0 g vs. 20.0 ± 1.1 g, respectively, Figure 9D and Table S2). During the experimental period, the survival rate of the experimental animals was 100%, suggesting that treatment with cepharanthine did not cause drug toxicity to the host. Moreover, the cepharanthine-treated groups presented more obviously sparse tumor cellularity and apoptosis than did the vehicle-treated control group. Similar to the in vitro analysis results, cepharanthine at 15 mg/kg and 30 mg/kg significantly reduced ki67 protein expression and increased the protein expression of cleaved caspase-3, phospho-AMPK, phospho-p53, and Nrf2 in C33A xenograft tumor mice compared with the vehicle-treated control group (ki67, 66.8 ± 8.4 vs. 34.1 ± 4.9 vs. 14.8 ± 4.8 positive cells; cleaved caspase-3, 2.54 ± 0.72 vs. 12.7 ± 2.6 vs. 27.0 ± 12.3 positive cells; phospho-AMPK, 1.92 ± 1.06 vs. 12.2 ± 4.9 vs. 17.2 ± 5.2 positive cells; phospho-p53, 13.1 ± 3.0 vs. 70.0 ± 14.9 vs. 112.8 ± 20.8 positive cells; Nrf2, 1.45 ± 1.19 vs. 3.95 ± 2.04 vs. 34.0 ± 10.8 positive cells, respectively, Figure 10 and Table S2, *p* < 0.05). These results further suggested that cepharanthine treatment can inhibit the growth of C33A xenograft tumors by targeting the AMPK/p53 and Nrf2 pathways and activating apoptosis in vivo.

## 4. Discussion

**Figure 11:**
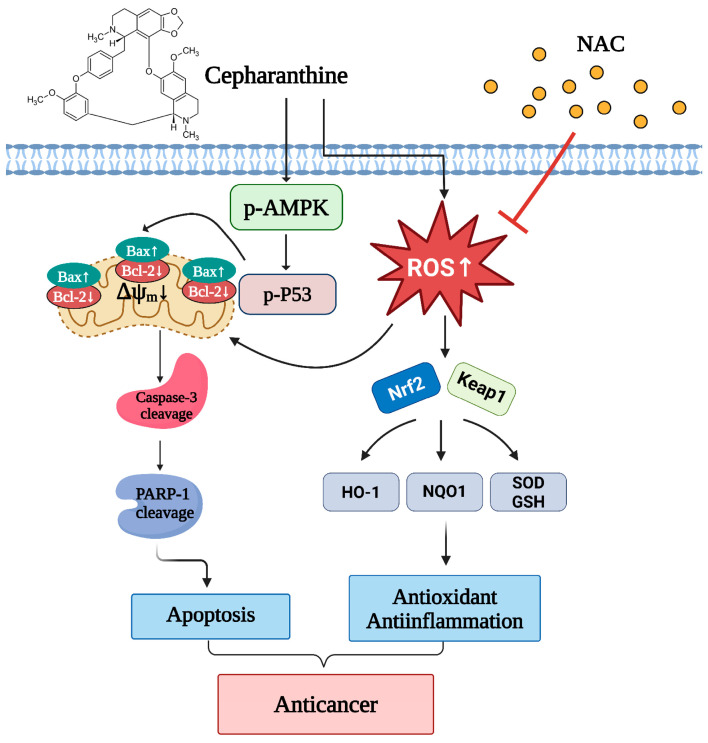
Schematic diagram of the anticancer mechanism of cepharanthine in cervical cancer through the AMPK/P53 and Nfr2/Keap1 pathways. Cepharanthine has an anticancer effect on human cervical cancer cells that is dependent on mitochondria-mediated intrinsic apoptosis through the modulation of the phosphorylating-AMPK/P53 pathway, upregulation of Bax expression, downregulation of Bcl-2 expression and induction of ROS production. Concurrently, cepharanthine increased ROS accumulation to induce oxidative stress by regulating the Nrf2/Keap1 pathway. NAC, an ROS scavenger, inhibited the ROS accumulation and reversed the apoptotic cell death of cepharanthine-treated cervical cancer cells. The chart drawing was performed using https://ailog.tw/lifelog/2021/06/10/picture-manager/ (accessed on 18 April 2025).

In this study, we employed both in vitro and in vivo models to evaluate whether cepharanthine can suppress cell viability and induce mitochondrial apoptosis in cervical cancer cells (CaSki, HeLa, and C33A) through AMPK/p53 pathway activation and Nrf2/Keap1 pathway modulation (Figure 11). Our findings suggest that cepharanthine could represent a novel therapeutic approach for the treatment of cervical cancer.

Our study revealed that, for each dose and treatment duration (25 μM and 50 μM; 24 and 48 h, respectively), cepharanthine strongly reduced cell viability and colony formation, particularly in CaSki and HeLa cells (Figure 1B–F). In addition, cepharanthine treatment clearly inhibited cervical cancer cell-derived xenograft animal tumor growth (Figure 9). These results were consistent with those of previous studies reported by Yang et al. [26], Su et al. [27], and Dong et al. [28], who reported that cepharanthine has a higher ability to inhibit nasopharyngeal carcinoma, colorectal cancer (SW480, SW620, and LoVo) and prostate cancer (PC-3 and DU145) growth. In addition, cepharanthine had a more significant cytotoxic effect on CaSki and HeLa than C33A cells, which may be due to the CaSki and HeLa cells being infected with HPV types 16 and 18, respectively [29]. The C-33 A cells were HPV-negative cells that expressed the oncogenes p53 or pRb protein [30].

Furthermore, our findings revealed that cepharanthine triggers apoptosis in cervical cancer cells, as evidenced through DAPI staining (cellular fluorescence) and qualitative flow cytometry analysis (Figure 2), along with Western blot analysis, which revealed elevated Bax/Bcl-2 ratios, increased levels of cleaved PARP-1 and cleaved caspase 3 proteins, and increased AMPK/p53 phosphorylation (Figure 6). The results align with the findings previously documented by Fang et al. [31] and Liang et al. [32], who reported that cepharanthine could facilitate apoptosis in a cervical cancer cell line (HeLa) and hepatocellular carcinoma cell lines (HepG2 and SMMC-7721), which activated caspase-3 and inactivated JAK2/STAT3 phosphorylation, even upregulating p53 and Bax in HNSCCs and melanoma [33,34]. Kiyomi et al. [35] also indicated that a higher concentration of cepharanthine induced cell death through late apoptosis, while a lower concentration caused early apoptotic cell death in breast cancer cells (MDA-MS-231 and MCF-7) and not necrosis. In addition to promoting apoptosis, Li et al. [36] reported that cepharanthine could induce autophagy to promote cell death by increasing p38 phosphorylation in lung adenocarcinoma cells (A549) and that cepharanthine was a novel ferroptosis-inducing agent. These different processes increase the sensitivity of cancer cells to cepharanthine treatment and more efficiently inhibit tumor growth and metastasis.

As reported in early studies, cepharanthine induced cell death by causing cell cycle arrest at the sub-G1 phase (HT-29) [24] or G0/G1 phase (MGF-7, MDA-MB-231, Hep3B, HCCLM3, AGS, HGC27, MCF, and HT-29) [11,14,22,24]. In this study, cepharanthine also caused sub-G1 phase arrest in HeLa and C33A cells, and a greater percentage of CaSki cells were in the G0/G1 phase (Figure 3). These differences may be associated with the cancer cell type specificity (doubling time: 23–25 h for HeLa cells, 23–35 h for C33A cells, 24–26 h for CaSki cells) [37] and the dose- or time-dependent bioavailability of cepharanthine. However, several studies have revealed that cepharanthine treatment significantly reduces mitochondrial ∆ψm and increases mitROS and intracellular ROS levels to increase oxidative stress damage, resulting in cancer apoptosis or ferroptosis in some common cancers, including breast (MDA-MB-231 and BT549) [17], lung (A549 and Lewis) [38], and prostate (LNCap and 22Rv1) [39]. Bai et al. [40] and Li et al. [41] reported that cepharanthine can destroy mitochondria, thereby inhibiting the antioxidative function of Nrf2 to promote ROS generation and weaken the antioxidant agents catalase (CAT) and superoxide dismutase (SOD) in lung and colorectal cancers (SW480 and SW620). Selvaraj et al. [42] reported that antioxidant-reducing molecules such as glutathione (GSH), the antioxidant enzyme SOD, and thioredoxin (TDX) are deficient in common cancers because of mitochondrial- and ROS-induced oxidative stress. The results of the above studies were consistent with our findings that cepharanthine treatment significantly reduces mitochondrial ∆ψm, increases ROS to reduce the T-GSH/GSSG ratio, and significantly inhibits SOD activity and Nrf2 protein expression in CaSki and HeLa cells (Figure 5 and Figure 7), whereas the T-GSH/GSSG ratio and Nrf2 levels do not decrease in C33A cells. Lu et al. [22] reported that cepharanthine increases ROS and oxidative stress levels by activating Nrf2 and inactivating SOD2 protein expression, resulting in anti-gastric cancer effects (AGS and HGC27). Similarly, Zhao et al. [43] revealed that magnolol protects against enterovirus 71 (EV71) by activating Nfr2, facilitating the SLC7A11-mediated GSH axis to increase intracellular GSH and GSH/GSSG levels and neutralize increased intracellular ROS in cancer therapy [44]. Lee et al. also reported that piperlongumine treatment could activate the Nrf2/HO-1 pathway to induce apoptosis in breast cancer (MCF-7 and MCF-10A), increasing the GSH/GSSG ratio and ROS generation only in MCF-7 cells; MCF-10A cells did not show significant changes [45]. Moreover, cepharanthine co-treatment with the ROS scavenger NAC can significantly inhibit intracellular ROS levels and reverse cepharanthine-induced apoptosis in CaSki, HeLa, and C33A cells (Figure 8).

In addition, Ekiner et al. [46] and Al-Mubarak et al. [47] also revealed that Nfr2 activation is regulated in the canonical Keap1-dependent and non-canonical Keap1-independent pathways. Our data revealed that cepharanthine treatment induced oxidative stress to disrupt the Nrf2-Keap1 complex and prevent Nrf2 degradation in C33A cells, whereas Keap1 uncouples from Nrf2, resulting in Nrf2 being ubiquitinated and degraded in CaSki and HeLa cells, while the Nrf2 downstream target enzymes NQO1 and HO-1 are also involved in antioxidant defense and immune modulation [48,49]. These differences indicate that the cepharanthine-mediated Nrf2-Keap1 pathway may be dependent on cell specificity and associated with the intensity and duration of oxidative stress.

## 5. Conclusions

Our findings demonstrate that cepharanthine significantly induces mitochondrial apoptosis in human cervical cancer cells both in vitro and in vivo, as evidenced by the increased expression of cleaved PARP-1 and cleaved caspase-3 proteins, along with an elevated Bax/Bcl-2 ratio. Additionally, cepharanthine disrupts mitochondrial integrity and enhances ROS production, thereby inducing oxidative stress, which activates the AMPK/p53 pathway and modulates the Nrf2/Keap1 pathway in cervical cancer cells. Moreover, cepharanthine has a suppressive effect on tumor growth in BALB/c nude mice, with minimal or no cytotoxic effects, which highlights cepharanthine as a promising therapeutic candidate for cervical cancer treatment.

## Data Availability

Research data and analyses can be obtained from the corresponding author upon valid request.

## Supplementary Materials

The following supporting information can be downloaded at https://www.mdpi.com/article/10.3390/antiox14111324/s1, Table S1. Comparison of the variations in experiments across cervical cancer cells. Table S2. Comparison of the variations in experiments across cervical cancer cell-derived xenograft nude mice.
