# Supplementary material for: Cepharanthine Induces Oxidative Stress and Apoptosis in Cervical Cancer via the Nrf2/Keap1 Pathway
Source: Antioxidants (Basel). 2025 Nov 1;14(11):1324. doi: 10.3390/antiox14111324 (PMC12649129; doi:10.3390/antiox14111324)
Supplement: Supplementary file 1 [file antioxidants-14-01324-s001.zip › antioxidants-3848807-supplementary.pdf]

Table S1. Comparision of the variations in experiment across cervical cancer cells

Experiment	Cell line	Mean ± SD	F(*df*)	Con vs C25 *p-value*	Con vs C50 *p-value*	C25 vs C50 *p-value*
**Cell Viability, 24h**	CaSki	Con = 100 ± 0.00, C25 = 75.17 ± 2.09, C50 = 26.90 ± 2.38	F(2,6) = 1241.58	< 0.0001	< 0.0001	< 0.0001
	HeLa	Con = 100 ± 0.00, C25 = 54.81 ± 0.86, C50 = 17.38 ± 0.68	F(2,6) = 11891.51	< 0.0001	< 0.0001	< 0.0001
	C33A	Con = 100 ± 0.00, C25 = 79.79 ± 3.73, C50 = 63.01 ± 1.11	F(2,6) = 204.25	0.0001	< 0.0001	0.0002
**Cell Viability, 48h**	CaSki	Con = 100 ± 0.00, C25 = 39.26 ± 1.02, C50 = 14.14 ± 2.28	F(2,6) = 2974.45	< 0.0001	< 0.0001	< 0.0001
	HeLa	Con = 100 ± 0.00, C25 = 26.28 ± 0.90, C50 = 13.19 ± 2.34	F(2,6) = 3143.65	< 0.0001	< 0.0001	0.0001
	C33A	Con = 100 ± 0.00, C25 = 42.81 ± 2.96, C50 = 35.53 ± 0.98	F(2,6) = 1158.96	< 0.0001	< 0.0001	0.0061
**Colony Formation**	CaSki	Con = 100 ± 0.00, C25 = 49.90 ± 6.61, C50 = 8.30 ± 1.36	F(2,6) = 416.63	< 0.0001	< 0.0001	< 0.0001
	HeLa	Con = 100 ± 0.00, C25 = 58.54 ± 5.88, C50 = 1.07 ± 0.28	F(2,6) = 640.92	< 0.0001	< 0.0001	< 0.0001
	C33A	Con = 100 ± 0.00, C25 = 51.67 ± 3.66, C50 = 1.86 ± 0.93	F(2,6) = 1517.09	< 0.0001	< 0.0001	< 0.0001
**DAPI**	CaSki	Con = 14.53 ± 6.02, C25 = 34.95 ± 7.31, C50 = 63.15 ± 3.48	F(2,15) = 105.47	0.0001	< 0.0001	< 0.0001
	HeLa	Con = 4.88 ± 2.12, C25 = 29.24 ± 11.59, C50 = 72.22 ± 26.35	F(2,24) = 37.68	0.013	< 0.0001	< 0.0001
	C33A	Con = 1.47 ± 1.67, C25 = 38.91 ± 11.57, C50 = 50.12 ± 12.06	F(2,24) = 62.14	< 0.0001	< 0.0001	0.055
**Apotosis**	CaSki	Con = 11.08 ± 1.34, C25 = 16.48 ± 0.17, C50 = 19.08 ± 3.91	F(2,9) = 11.69	0.0265	0.0027	0.3186
	HeLa	Con = 6.81 ± 1.78, C25 = 24.82 ± 8.14, C50 = 77.28 ± 3.34	F(2,24) = 448.7	<0.0001	<0.0001	<0.0001
	C33A	Con = 5.50 ± 1.10, C25 = 10.88 ± 3.14, C50 = 24.08 ± 3.98	F(2,9) = 40.81	0.074	< 0.0001	0.0004
**Cell cycle**	CaSki					
	SubG1	Con = 0.62 ± 0.13, C25 = 0.71 ± 0.10, C50 = 0.94 ± 0.16	F(2,6) = 3.88	0.7889	0.0814	0.1909
	G0G1	Con = 45.46 ± 1.78, C25 = 74.67 ± 1.21, C50 = 58.24 ± 1.21	F(2,6) = 316.82	< 0.0001	0.0001	< 0.0001
	S	Con = 17.67 ± 0.27, C25 = 6.93 ± 0.18, C50 = 3.62 ± 0.20	F(2,6) = 3187.59	< 0.0001	< 0.0001	< 0.0001
	G2M	Con = 35.79 ± 2.50, C25 = 16.50 ± 1.02, C50 = 36.42 ± 1.81	F(2,6) = 109.46	< 0.0001	0.9113	< 0.0001
	HeLa					
	SubG1	Con = 2.64 ± 0.86, C25 = 3.93 ± 0.54, C50 = 67.99 ± 0.34	F(2,6) = 10990.74	0.0949	< 0.0001	< 0.0001
	G0G1	Con = 66.53 ± 0.17, C25 = 79.87 ± 0.42, C50 = 15.49 ± 1.18	F(2,6) = 6538.72	< 0.0001	< 0.0001	< 0.0001
	S	Con = 10.00 ± 0.55, C25 = 5.32 ± 0.42, C50 = 9.54 ± 0.39	F(2,6) = 94.93	< 0.0001	0.4868	0.0001
	G2M	Con = 19.07 ± 0.24, C25 = 10.24 ± 0.48, C50 = 7.49 ± 0.50	F(2,6) = 611.31	< 0.0001	< 0.0001	0.0005
	C33A					
	SubG1	Con = 0.34 ± 0.08, C25 = 0.68 ± 0.08, C50 = 35.42 ± 0.26	F(2,5) = 49055.28	0.0665	< 0.0001	< 0.0001
	G0G1	Con = 39.58 ± 0.94, C25 = 25.38 ± 0.67, C50 = 18.57 ± 0.85	F(2,5) = 440.83	< 0.0001	< 0.0001	0.0006
	S	Con = 18.84 ± 1.72, C25 = 24.22 ± 1.39, C50 = 20.13 ± 0.24	F(2,5) = 11.73	0.0124	0.6056	0.0537
	G2M	Con = 41.72 ± 3.63, C25 = 49.54 ± 0.82, C50 = 26.30 ± 0.19	F(2,5) = 58.87	0.0218	0.0019	0.0003
**Wound healing**	CaSki	Con = 33.68 ± 2.99, C25 = 66.41 ± 0.28, C50 = 95.23 ± 2.28	F(2,6) = 601.46	< 0.0001	< 0.0001	< 0.0001
	HeLa	Con = 26.46 ± 1.90, C25 = 51.50 ± 3.16, C50 = 93.23 ± 6.15	F(2,6) = 199.15	0.0008	< 0.0001	< 0.0001
	C33A	Con = 65.19 ± 1.47, C25 = 79.49 ± 3.02, C50 = 83.21 ± 3.82	F(2,6) = 31.52	0.0024	0.0007	0.334
**JC-1**	CaSki					
	polymers	Con = 94.34 ± 2.69, C25 = 95.06 ± 3.62, C50 = 67.14 ± 16.25	F(2,12) = 11.02	0.9938	0.0045	0.0038
	monomers	Con= 4.90 ± 1.67, C25 = 1.92 ± 1.02, C50 = 32.78 ± 15.53	F(2,12) = 13.15	0.8958	0.0033	0.0015
	HeLa					
	polymers	Con = 97.3 ± 1.08, C25 = 96.03 ± 0.37, C50 = 63.7 ± 21.04	F (2,6) = 7.347	0.9911	0.0342	0.0398
	monomers	Con = 2.7 ± 1.08, C25 = 3.96 ± 0.37, C50 = 36.3 ± 21.04	F (2,6) = 7.347	0.9911	0.0342	0.0398
	C33A					
	polymers	Con = 95.52 ± 2.83, C25 = 95.28 ± 1.64, C50 = 47.13 ± 12.48	F(2,15) = 82.66	0.9984	< 0.0001	< 0.0001
	monomers	Con = 4.33 ± 2.95, C25 = 4.35 ± 1.80, C50 = 52.85 ± 12.45	F(2,15) = 82.64	1	< 0.0001	< 0.0001

Continued Table S1. Comparision of the variations in experiment across cervical cancer cells

Experiment	Cell line	Mean ± SD	F(*df*)	Con vs C25 *p-value*	Con vs C50 *p-value*	C25 vs C50 *p-value*
**SOD**	CaSki	Con = 23.97 ± 2.68, C25 = 16.73 ± 0.83, C50 = 10.88 ± 1.58	F (2,6) = 37.14	0.0075	0.0003	0.0199
	HeLa	Con = 83.96 ± 7.09, C25 = 63.87 ± 16.45, C50 = 51.93 ± 16.41	F (2,6) = 3.997	0.2619	0.07	0.5799
	C33A	Con = 107.4 ± 4.05, C25 = 76.99 ± 3.02, C50 = 66.11 ± 4.28	F (2,6) = 93.60	0.0002	<0.0001	0.0305
**T-GSH/ GSSG**	CaSki	Con = 2.11 ± 0.60, C25 = 1.63 ± 0.17, C50 = 1.21 ± 0.33	F (2,15) = 7.242	0.1386	0.0046	0.2129
	HeLa	Con = 4.80 ± 0.79, C25 = 4.64 ± 1.09, C50 = 1.44 ± 0.28	F (2,12) = 28.24	0.9467	<0.0001	0.0001
	C33A	Con = 1.35 ± 0.35, C25 = 3.39 ± 1.15, C50 = 3.06 ± 0.57	F (2,12) = 10.18	0.0032	0.0109	0.7753
**ROS**	CaSki	Con = 1.00 ± 0.00, C25 = 2.63 ± 0.24, C50 = 6.30 ± 1.52	F(2,15) = 55.99	0.0162	<0.0001	<0.0001
	HeLa	Con = 1.00 ± 0.00, C25 = 2.27 ± 0.53, C50 = 7.47 ± 0.82	F(2,15) = 223.8	0.0037	<0.0001	<0.0001
	C33A	Con = 1.00 ± 0.00, C25 = 1.98 ± 0.45, C50 = 4.53 ± 2.02	F(2,12) = 11.63	0.424	0.0014	0.0141
**Western Blot**						
**PARP-1**	CaSki	Con = 1.00 ± 0.00, C25 = 1.32 ± 0.34, C50 =1.87 ± 0.47	F(2,42) = 25.57	0.0332	<0.0001	0.0002
	HeLa	Con = 1.00 ± 0.00, C25 = 2.27 ± 0.66, C50 = 6.73 ± 1.61	F(2,57) = 179.61	0.0005	<0.0001	<0.0001
	C33A	Con = 1.00 ± 0.00, C25 = 2.15 ± 0.40, C50 = 3.88 ± 1.86	F (2,30) = 19.12	0.0503	<0.0001	0.0025
**Bcl-2**	CaSki	Con = 1.00 ± 0.00, C25 = 0.91 ± 0.15, C50 = 0.56 ± 0.24	F(2,33) = 24.23	0.4062	<0.0001	<0.0001
	HeLa	Con = 1.00 ± 0.00, C25 = 0.96 ± 0.05, C50 = 0.67 ± 0.19	F(2,42) = 36.69	0.5936	<0.0001	<0.0001
	C33A	Con = 1.00 ± 0.00, C25 = 0.55 ± 0.05, C50 = 0.47 ± 0.07	F(2,60) = 646.77	<0.0001	<0.0001	<0.0001
**Bax**	CaSki	Con = 1.00 ± 0.00, C25 = 1.16 ± 0.14, C50 = 1.74 ± 0.67	F(2,60) = 20.52	0.3737	<0.0001	<0.0001
	HeLa	Con = 1.00 ± 0.00, C25 = 1.1 ± 0.19, C50 = 2.11 ± 0.71	F(2,42) = 36.69	0.6986	<0.0001	<0.0001
	C33A	Con = 1.00 ± 0.00, C25 = 1.02 ± 0.13, C50 = 1.81 ± 0.54	F(2,51) = 36.49	0.962	<0.0001	<0.0001
**C-Cas-3**	CaSki	Con = 1.00 ± 0.00, C25 = 1.35 ± 0.53, C50 = 4.05 ± 0.94	F(2,72) = 177.8	0.1209	<0.0001	<0.0001
	HeLa	Con = 1.00 ± 0.00, C25 = 2.72 ± 0.51, C50 = 9.51 ± 1.86	F(2,72) = 409.61	<0.0001	<0.0001	<0.0001
	C33A	Con = 1.00 ± 0.00, C25 = 1.33 ± 0.14, C50 = 3.11 ± 0.73	F(2,45) = 110.8	0.0833	<0.0001	<0.0001
**p-APMK**	CaSki	Con = 1.00 ± 0.00, C25 = 0.24 ± 0.06, C50 = 0.81 ± 0.10	F(2,21) = 316	<0.0001	<0.0001	<0.0001
	HeLa	Con = 1.00 ± 0.00, C25 = 0.47 ± 0.11, C50 = 1.23 ± 0.12	F(2,18) = 115.73	<0.0001	0.0009	<0.0001
	C33A	Con = 1.00 ± 0.00, C25 = 0.95 ± 0.03, C50 = 16.10 ± 0.78	F (2,6) = 1136	0.9884	<0.0001	<0.0001
**p-p53**	CaSki	Con = 1.00 ± 0.00, C25 = 0.72 ± 0.11, C50 = 0.62 ± 0.03	F(2,33) = 108.4	<0.0001	<0.0001	0.0036
	HeLa	Con = 1.00 ± 0.00, C25 = 0.89 ± 0.08, C50 = 1.21 ± 0.13	F(2,33) = 38.38	0.0173	<0.0001	<0.0001
	C33A	Con = 1.00 ± 0.00, C25 = 1.06 ± 0.19, C50 = 2.09 ± 0.39	F(2,33) = 72.27	0.8019	<0.0001	<0.0001
**Nrf-2**	CaSki	Con = 1.00 ± 0.00, C25 = 0.75 ± 0.18, C50 = 0.51 ± 0.18	F(2,51) = 52.03	< 0.0001	< 0.0001	< 0.0001
	HeLa	Con = 1.00 ± 0.00, C25 = 0.88 ± 0.10, C50 = 0.54 ± 0.14	F(2,51) = 101.82	0.0027	< 0.0001	< 0.0001
	C33A	Con = 1.00 ± 0.00, C25 = 1.12 ± 0.07, C50 = 2.74 ± 0.96	F(2,36) = 40.36	0.8486	< 0.0001	< 0.0001
**Keap-1**	CaSki	Con = 1.00 ± 0.00, C25 = 0.26 ± 0.06, C50 = 0.22 ± 0.07	F(2,66) = 1684.11	< 0.0001	< 0.0001	0.006
	HeLa	Con = 1.00 ± 0.00, C25 = 0.25 ± 0.09, C50 = 0.18 ± 0.07	F(2,66) = 1066.26	< 0.0001	< 0.0001	0.0022
	C33A	Con = 1.00 ± 0.00, C25 = 0.64 ± 0.11, C50 = 0.43 ± 0.10	F(2,66) = 255.95	< 0.0001	< 0.0001	< 0.0001
**NQO-1**	CaSki	Con = 1.00 ± 0.00, C25 = 0.70 ± 0.12, C50 = 0.65 ± 0.21	F (2,45) = 28.67	< 0.0001	<0.00001	0.6029
	HeLa	Con = 1.00 ± 0.00, C25 = 0.94 ± 0.07, C50 = 0.68 ± 0.13	F (2,45) = 64.59	0.1015	< 0.0001	< 0.0001
	C33A	Con = 1.00 ± 0.00, C25 = 0.76 ± 0.16, C50 = 0.59 ± 0.12	F (2,45) = 50.36	< 0.0001	< 0.0001	0.0006
**HO-1**	CaSki	Con = 1.00 ± 0.00, C25 = 2.19 ± 0.52, C50 = 1.19 ± 0.43	F(2,60) = 56.3	< 0.0001	0.2653	< 0.0001
	HeLa	Con = 1.00 ± 0.00, C25 = 1.61 ± 0.35, C50 = 0.51 ± 0.11	F(2,60) = 144.69	< 0.0001	< 0.0001	< 0.0001
	C33A	Con = 1.00 ± 0.00, C25 = 2.56 ± 0.26, C50 = 4.85 ± 1.81	F(2,60) = 71.01	< 0.0001	< 0.0001	< 0.0001

Continued Table S1. Comparision of the variations in experiment across cervical cancer cells

Experiment	Cell line	Mean ± SD	F(*df*)	Con vs. C50 *p-value*	Con vs. C50+NAC1 *p-value*	Con vs. C50+NAC2 *p-value*	C50 vs. C50+NAC1 *p-value*	C50 vs. C50+NAC2 *p-value*
**Cep + NAC_ROS**	CaSki	Con = 1.00 ± 0.00, C50 = 12.11 ± 2.16, C50+NAC1 = 10.28 ± 1.19, C50+NAC2 = 10.11 ± 1.47	F(3,8) = 36.27	<0.0001	0.0002	0.0003	0.4474	0.378
	HeLa	Con = 1.00 ± 0.00 C50 = 9.39 ± 0.66, C50+NAC1 = 7.39 ± 0.81, C50+NAC2 = 5.31 ± 0.67	F(3,8) = 100.8	<0.0001	<0.0001	0.0001	0.0176	0.0002
**Cep + NAC_Apotosis**	CaSki	Con = 8.37 ± 3.39, C50 = 45.15 ± 1.20, C50+NAC1 = 34.43 ± 0.75, C50+NAC2 = 33.2 ± 0.80	F(3,8) = 205.9	<0.0001	<0.0001	<0.0001	0.0005	0.0002
	HeLa	Con = 6.55 ± 0.18, C50 = 29.10 ± 1.94, C50+NAC1 = 16.98 ± 1.13, C50+NAC2 = 15.77 ± 3.00	F(3,8) = 72.93	<0.0001	0.0006	0.0014	0.0002	0.0001

Experiment	Cell line	Mean ± SD	F(*df*)	Con vs. C50 *p-value*	Con vs. C50+NAC3 *p-value*	Con vs. C50+NAC5 *p-value*	C50 vs. C50+NAC3 *p-value*	C50 vs. C50+NAC5 *p-value*
**Cep + NAC_ROS**	C33A	Con = 1.00 ± 0.00, C50 = 11.01 ± 4.25, C50+NAC3 = 8.51 ± 0.59, C50+NAC5 = 4.33 ± 1.36	F (3,8) = 11.64	0.0027	0.0148	0.3351	0.5568	0.0275
**Cep + NAC_Apotosis**	C33A	Con = 2.62 ± 1.58, C50 = 71.08 ± 8.55, C50+NAC3 = 5.08 ± 1.37, C50+NAC5 = 3.30 ± 1.13	F(3,8) = 173.3	<0.0001	0.9015	0.9974	<0.0001	<0.0001

Table S2. Comparision of the variations in experiment across cervical cancer cell-derived xenograft nude mice

Experiment	Cell line	Mean ± SD	F(*df*)	Con vs C15 *p-value*	Con vs C30 *p-value*	C15 vs C30 *p-value*
**Animal**	Tumor Volume (25 d)	Con = 944.47 ± 289.20, C15 = 473.29 ± 206.25, C30 = 339.15 ± 197.59	F(2,21) = 12.85	0.0032	0.0003	0.5429
	Tumor Weight (25 d)	Con = 448.13 ± 165.94, Cep15 = 232.75 ± 77.53, Cep30 = 200.38 ± 95.06	F(2,21) = 8.94	0.0076	0.0023	0.8681
	Body Weight (25 d)	Con = 20.00 ± 1.10, Cep15 = 20.00 ± 0.63, Cep30 = 19.80 ± 0.98	F(2,21) = 0.57	0.9643	0.7245	0.568
**Immunohistochemistry**	Ki-67	Con = 66.75 ± 8.36, C15 = 34.13 ± 4.94, C30 = 14.75 ± 4.86	F (2,69) = 421.8	<0.0001	<0.0001	<0.0001
	C-Cas-3	Con = 2.54 ± 0.72, C15 = 12.67 ± 2.55, C30 = 27.00 ± 12.26	F (2,69) = 69.10	<0.0001	<0.0001	<0.0001
	p-AMPK	Con = 1.92 ± 1.06, C15 = 12.17 ± 4.86, C30 = 17.17 ± 5.17	F (2,69) = 84.50	<0.0001	<0.0001	0.0002
	p-p53	Con = 13.05 ± 2.95, C15 = 70.00 ± 14.94, C30 = 112.82 ± 20.77	F (2,63) = 249.2	<0.0001	<0.0001	<0.0001
	Nrf-2	Con = 1.45 ± 1.19, C15 = 3.95 ± 2.04, C30 = 34.00 ± 10.77	F (2,57) = 161.9	0.4338	<0.0001	<0.0001
